# Supplementary figures and images for: Aglianico Grape Seed Semi-Polar Extract Exerts Anticancer Effects by Modulating MDM2 Expression and Metabolic Pathways
Source: Cells. 2023 Jan 4;12(2):210. doi: 10.3390/cells12020210 (PMC9856309; doi:10.3390/cells12020210)

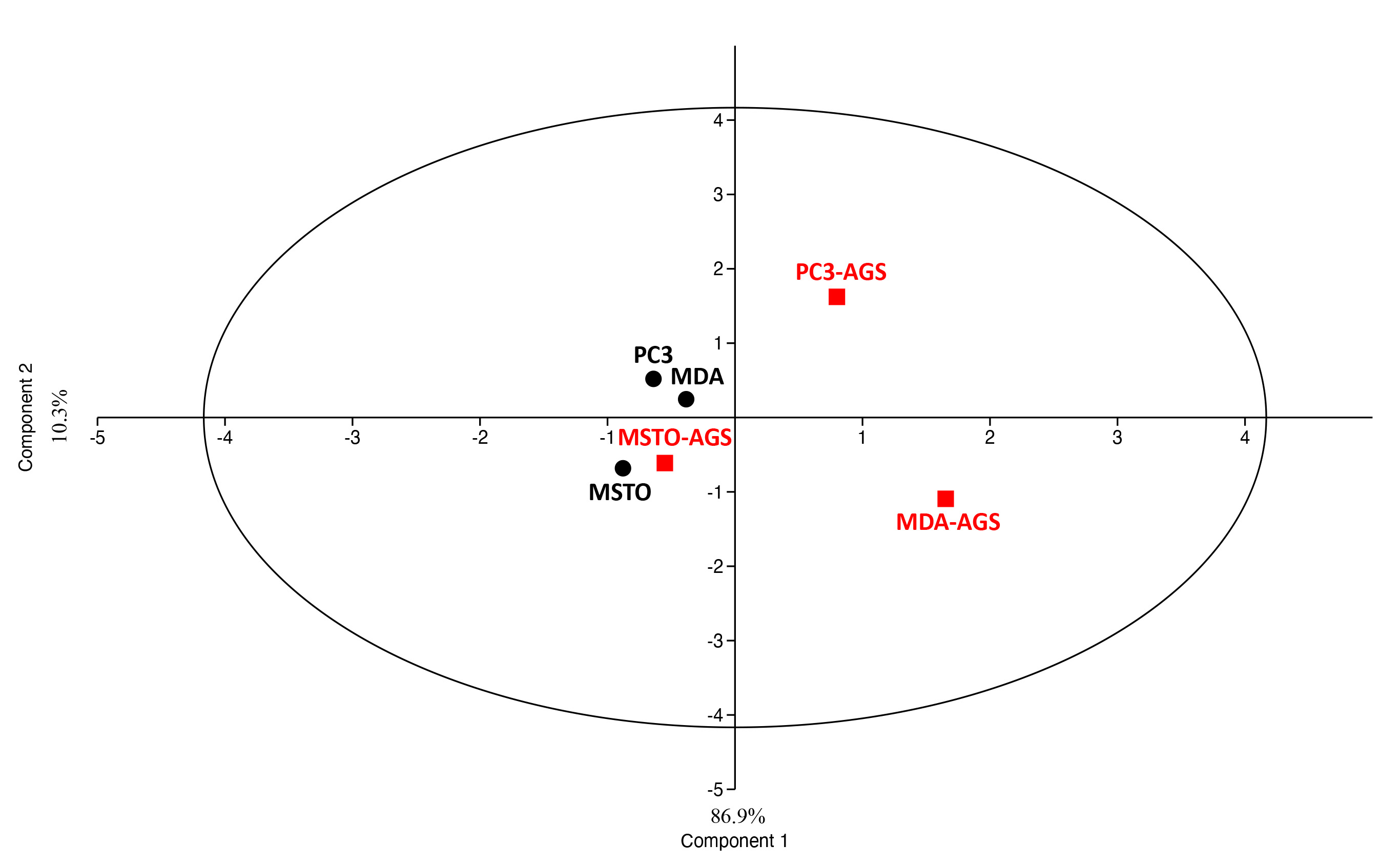

Supplement: Supplementary file 1 [file cells-12-00210-s001.zip › Figure S1.tif]

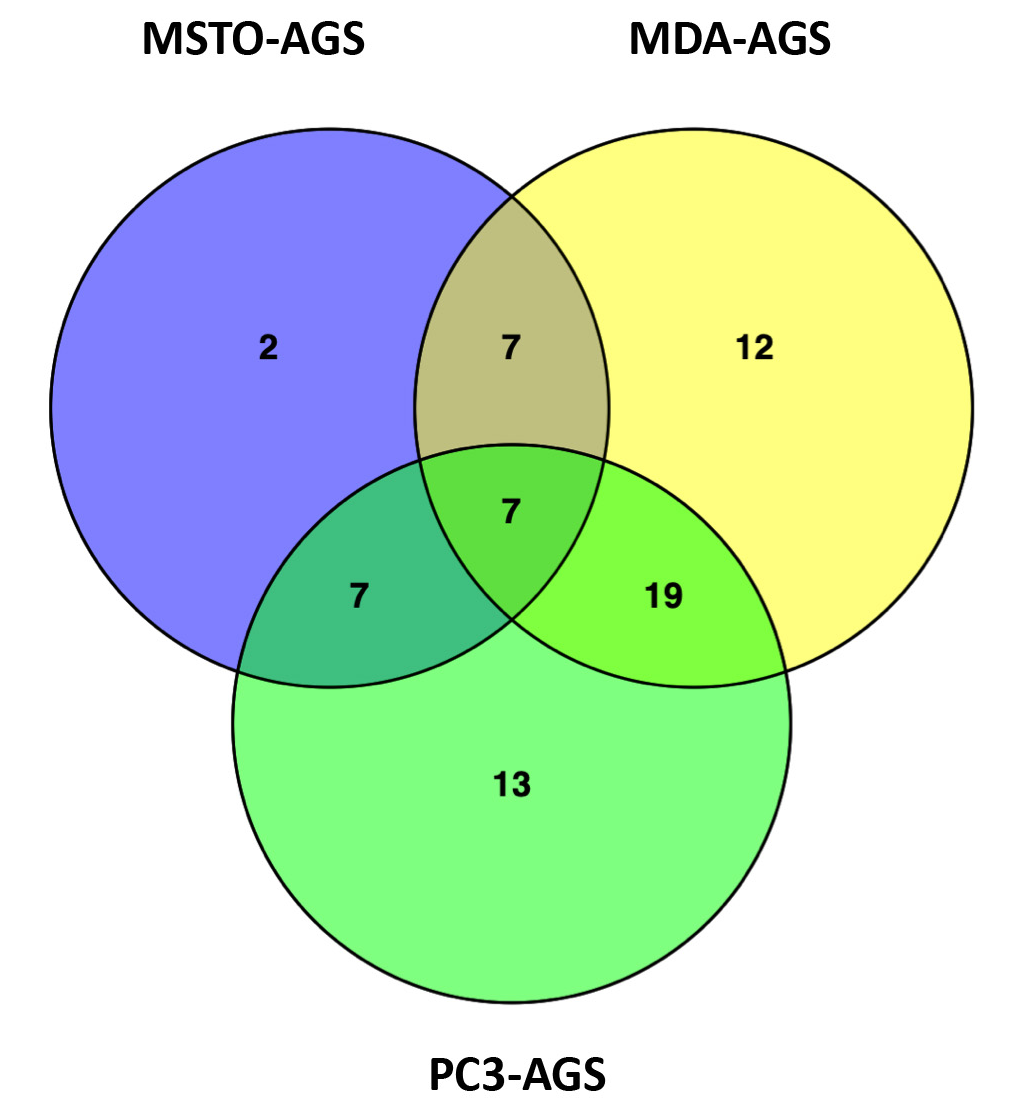

Supplement: Supplementary file 1 [file cells-12-00210-s001.zip › Figure S2.tif]
